# Second-generation supraglottic airway in laparoscopic donor nephrectomy

**DOI:** 10.1038/s41598-023-34691-x

**Published:** 2023-05-24

**Authors:** Ja Eun Lee, Ha Yeon Kim, Kyo Won Lee, Gaab Soo Kim

**Affiliations:** 1 Department of Anesthesiology and Pain Medicine, Samsung Medical Center, Sungkyunkwan University School of Medicine, Seoul, Korea; 2 Department of Anesthesiology and Pain Medicine, Ajou University School of Medicine, Suwon, Korea; 3 Department of Surgery, Samsung Medical Center, Sungkyunkwan University School of Medicine, Seoul, Korea

**Keywords:** Kidney diseases, Oral anatomy

## Abstract

Supraglottic airway (SGA) may have advantages over endotracheal tube (ETT) regarding laryngospasm, coughing, sore throat, and hemodynamic changes; however, studies on the use of SGA in laparoscopic donor nephrectomy (LDN) are lacking. Here, we aimed to confirm the safety and feasibility of second-generation SGA in LDN and compare them with those of ETT. Enrolled adult donors (aged > 18 years) who underwent LDN between August 2018 and November 2021 were divided into two groups—ETT vs. SGA. Airway pressure, lung compliance, desaturation, and hypercapnia were recorded during surgery. After propensity score matching for baseline characteristics and surgical duration, 82 and 152 donors were included in the ETT and SGA groups, respectively, and their outcomes were compared. The peak airway pressure was lower in the SGA group than in the ETT group 5 min after pneumoperitoneum. Dynamic lung compliance was higher in the SGA group than in the ETT group during surgery. There were no cases of intraoperative desaturation, hypercapnia, or postoperative aspiration pneumonitis. The use of second-generation SGA, a safe alternative to ETT for LDN, resulted in reduced airway resistance and increased lung compliance, which suggests its benefits for airway management in kidney donors.

## Introduction

Although endotracheal intubation is the gold standard for maintaining the airway under general anesthesia, it can induce physiological stress responses. The use of supraglottic airway (SGA) is known to reduce the incidence of laryngospasm, coughing, sore throat, and hemodynamic changes compared to that of endotracheal tube (ETT)^1,2^. Because of these advantages, the use of SGA has gradually increased. However, the risk of inadequate ventilation and pulmonary aspiration remains a major concern associated with the use of SGA^2^.

As living kidney donors have no physical benefit from donation, living-donor nephrectomy aims to enhance donor recovery with safe organ acquisition^3^. As one of the enhanced recovery protocols for donors, the laparoscopic approach is favored over the open approach because it involves reduced postoperative pain and shorter hospital stay and helps the donors resume normal activities quickly^4,5^. SGA has advantages in terms of enhanced donor recovery because it can reduce stress responses compared to ETT. However, the use of SGA has been limited in laparoscopic donor nephrectomy (LDN) because of concerns regarding inadequate ventilation and gastric regurgitation in the lateral position and pneumoperitoneum. Although many studies have demonstrated that SGA is safe to use in laparoscopic surgeries^6–10^, they were mostly conducted under supine position, and the safety of SGA in lateral position under laparoscopy, a potentially unfavorable condition for SGA, still remains in question.

The design of SGA has been modified to achieve tight airway sealing and reduce gastric distension. The second-generation SGA provides higher sealing pressure than classical SGA and reduces the risk of aspiration by occluding the esophagus through SGA tip and providing gastric drainage channel^10^. In addition, some second-generation SGAs are known to reduce accidental rotation and displacement using buccal cavity stabilizer^11^. However, there is a lack of studies on the use of SGA in LDN. In our institute, second-generation SGA has been used in LDN since 2018. The aim of this study was to confirm the safety and feasibility of second-generation SGA in LDN and compare them with those of ETT.

## Methods

### Study design and population

This single-center retrospective study was approved by the Institutional Review Board of the Samsung Medical Center (2021-04-142). The Institutional Review Board of Samsung Medical Center waived the requirement for informed consent due to retrospective design of the study, and the study was conducted in accordance with the Declaration of Helsinki. Adult donors (> 18 years old) who underwent LDN between August 2018 and November 2021 were enrolled. Exclusion criteria were conversion to open surgery, violation of the anesthetic protocol, and failure of SGA insertion. Donors were divided into the ETT and SGA groups. Donor information was retrieved from electronic medical records.

### Study protocol

Anesthesia was induced with thiopental sodium (5 mg/kg) or propofol (2 mg/kg), vecuronium (0.1 mg/kg), and sevoflurane and maintained with desflurane. Neuromuscular blockade was monitored by Train-of-Four measurement with EZstim II (Life-Tech, Texas, the United States), and continuous vecuronium or rocuronium infusion was administered according to the attending anesthesiologist. The protocol for inducing anesthesia remained the same throughout the study period. Routine monitoring devices such as pulse oximetry, electrocardiography, and noninvasive blood pressure monitoring were used. Endotracheal intubation was performed with an internal diameter of 8.0 mm for male donors and 7.0 mm for female donors. The second-generation SGA, LMA Protector™ (Teleflex Medical Europe, Westmeath, Ireland) was chosen according to the manufacturer’s recommendations. The LMA Protector™ has two ports that serve for insertion of the gastric tube and suction of the laryngeal region. After SGA insertion, a 14 Fr gastric tube was inserted through the female port of the SGA. Mechanical ventilation using a 0.5 fraction of inspired oxygen at a fresh gas flow rate of 2 L/min was set with 5 to 6 cmH_2_O positive end-expiratory pressure (PEEP) and a tidal volume (TV) of 8 mL/kg of ideal body weight^12^. Respiratory rate was adjusted to maintain normocapnia. During pneumoperitoneum, the intra-abdominal carbon dioxide (CO_2_) pressure was adjusted to 12 mmHg.

### Data collection

Information about donor characteristics including age, sex, height, body weight, body mass index, American Society of Anesthesiologist physical status classification, and durations of anesthesia, surgery, and pneumoperitoneum was collected. The peak airway pressure (P_peak_), plateau airway pressure (P_plat_), PEEP, set TV (TV_set_), actual TV (TV_act_), pulse oximetry saturation (SpO_2_), end-tidal CO_2_ (EtCO_2_), and respiratory rate were recorded at five time points: after induction, after lateral position, 5 min after pneumoperitoneum, 5 min after pneumoperitoneum removal, and at the end of surgery. Dynamic compliance (C_dyn_) and static compliance (C_stat_) were calculated using Eqs. (1) and (2), respectively:

1 $$ {\text{C}}_{{{\text{dyn}}}} \left( {{\text{mL}}/{\text{cmH}}_{{2}} {\text{O}}} \right) \, = {\text{ TV}}_{{{\text{act}}}} \left( {{\text{mL}}} \right)/\left[ {{\text{P}}_{{{\text{peak}}}} \left( {{\text{cmH}}_{{2}} {\text{O}}} \right) \, {-}{\text{ PEEP }}\left( {{\text{cmH}}_{{2}} {\text{O}}} \right)} \right], $$

2 $$ {\text{C}}_{{{\text{stat}}}} \left( {{\text{mL}}/{\text{cmH}}_{{2}} {\text{O}}} \right) \, = {\text{ TV}}_{{{\text{act}}}} \left( {{\text{mL}}} \right)/\left[ {{\text{P}}_{{{\text{plt}}}} \left( {{\text{cmH}}_{{2}} {\text{O}}} \right) \, {-}{\text{ PEEP }}\left( {{\text{cmH}}_{{2}} {\text{O}}} \right)} \right]. $$

In the SGA group, the leakage fraction was calculated using Eq. (3):

3 $$ {\text{Leakage}}\,{\text{fraction }}\left( \% \right) \, = \, \left[ {\left[ {{\text{TV}}_{{{\text{set}}}} \left( {{\text{mL}}} \right) \, {-}{\text{ TV}}_{{{\text{act}}}} \left( {{\text{mL}}} \right)} \right]/{\text{TV}}_{{{\text{set}}}} \left( {{\text{mL}}} \right)} \right] \, \times { 1}00. $$

If TV_act_ was greater than TV_set_, the leakage fraction was regarded as zero. Data on the duration of postoperative care unit (PACU) stay, hospital stay, and postoperative pulmonary complications during hospital stay were collected.

### Statistical analysis

The primary outcomes were respiratory mechanics, including airway pressure and lung compliance. The secondary outcomes were the incidence of desaturation (SpO_2_ < 97%) and suboptimal ventilation (EtCO_2_ > 45 mmHg), leakage fraction in the SGA group, and incidence of postoperative pulmonary complications. To reduce the influence of potential confounding factors of baseline characteristics and surgical duration, propensity score matching was performed with nearest-neighbor matching without replacement. Each donor in the ETT group was matched to two donors in the SGA group (1:2 matching). The matching factors were age, sex, height, body weight, body mass index, anesthesia time, surgical time, and pneumoperitoneum time. Caliper size was defined as the 0.25 standard deviation of the logit of the propensity score^13^. Successful matching was determined using an absolute standardized difference < 0.2.

Continuous variables were expressed as mean ± standard deviation, and categorical variables as numbers and percentages. Continuous variables were compared using Student’s t-test, and categorical variables were compared using Fisher’s exact test or the chi-square test. Repeatedly measured variables were analyzed using the two-way mixed ANOVA test. When intergroup differences were statistically significant, post-hoc analyses were performed using the independent t-test for two samples, and the P-value was adjusted with Bonferroni correction for multiple comparisons. All statistical analyses were performed using IBM SPSS Statistics for Windows, version 25.0, (IBM Corporation, Armonk, NY, USA) or R software version 4.0.5. P < 0.05 was considered statistically significant.

## Results

**Figure 1:**
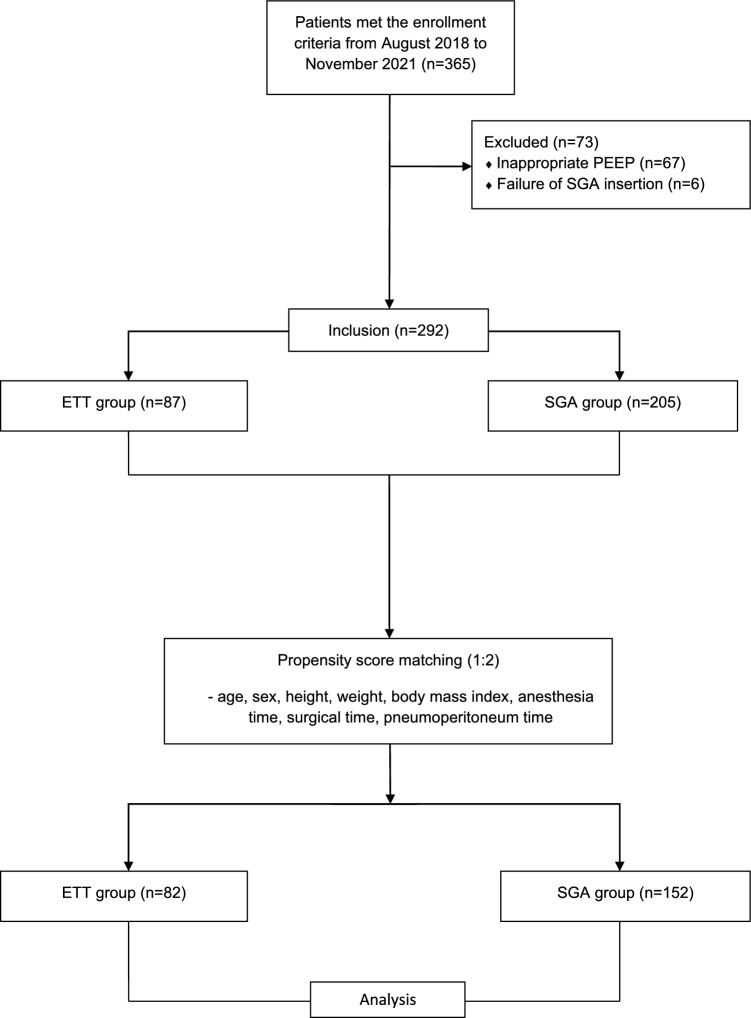
Flow diagram showing donor groups.

Between August 2018 and November 2021, 365 donors underwent LDN. Of these, 67 donors in the ETT group were excluded because of inadequate PEEP (0–4 mmHg). Failure of SGA insertion occurred in six donors who were excluded from the analysis. Finally, 292 donors were included in the analysis. After propensity score matching, 82 and 152 donors were included in the ETT and SGA groups, respectively (Fig. 1). The change in SGA size occurred in three donors in the SGA group. Failure of gastric tube insertion occurred in one donor in the SGA group.

Comparisons of preoperative and intraoperative variables before and after matching between the two groups are shown in Table 1. Before matching, anesthesia time and surgery time were unbalanced between the two groups, as demonstrated by the absolute standardized difference of > 0.2. After matching, heterogeneity was reduced, and all variables were within an absolute standardized difference of < 0.2.

**Table 1:** Comparison of the preoperative and intraoperative variables before and after matching.

	Before matching				After matching			
	ETT group (n = 87)	SGA group (n = 205)	P-value	SD	ETT group (n = 82)	SGA group (n = 152)	P-value	SD
Age, year	50 ± 12	50 ± 11	0.852	0.02	50 ± 12	50 ± 11	0.95	0.01
Sex, male (%)	39 (45)	84 (41)	0.631	0.08	38 (46)	66 (43)	0.771	0.06
Height, cm	163 ± 8	163 ± 8	0.95	0.01	50 ± 12	50 ± 11	0.95	0.01
Weight, kg	66 ± 11	64 ± 11	0.319	0.13	164 ± 9	163 ± 8	0.738	0.05
BMI, kg/m^2^	25 ± 3	25 ± 9	0.954	0.01	25 ± 3	24 ± 3	0.751	0.04
ASA I/II/III, n	44/41/2	102/102/1	0.348	0.03	40/40/2	73/78/1	0.499	0.03
Anesthesia time, min	239 ± 46	221 ± 37	0.002	0.43	232 ± 37	228 ± 37	0.382	0.12
Surgery time, min	187 ± 47	169 ± 37	0.002	0.43	180 ± 35	175 ± 37	0.364	0.13
PP time, min	114 ± 28	108 ± 35	0.121	0.19	114 ± 28	111 ± 36	0.481	0.09

Data are presented as mean ± standard deviation or frequency (%).

*SD* standardized difference, *BMI* body mass index, *PP* pneumoperitoneum.

**Figure 2:**
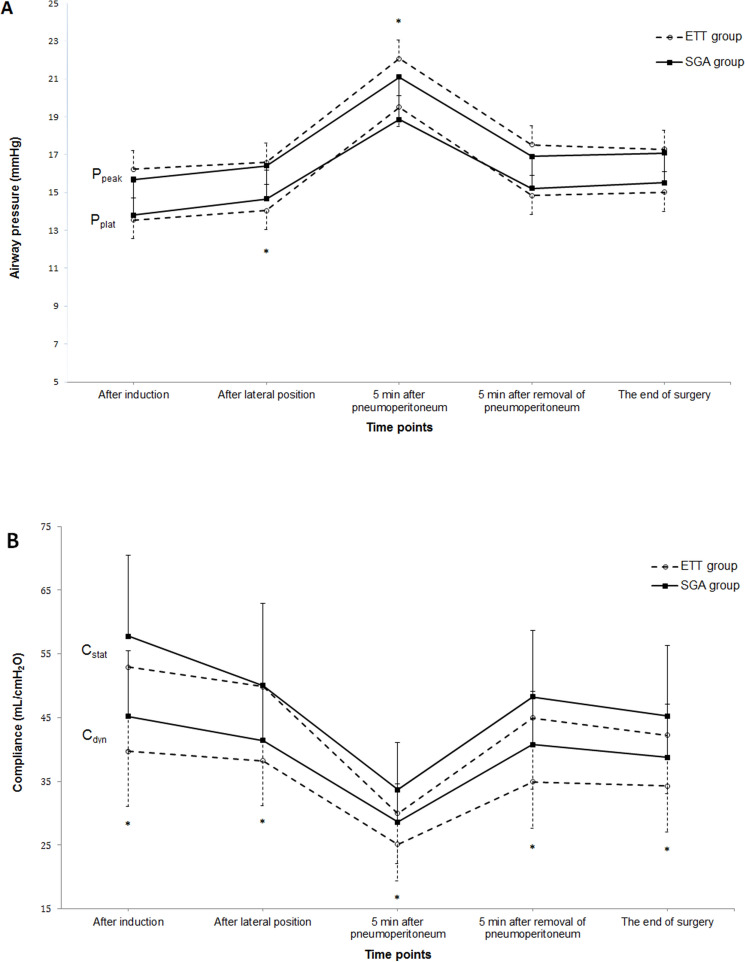
Changes in airway pressure during surgery. Data are presented as mean ± standard deviation. *ETT group* endotracheal tube group, *SGA group* supraglottic airway group, *P*_*peak*_ peak airway pressure, *P*_*plat*_ plateau airway pressure, *C*_*stat*_ static compliance, *C*_*dyn*_ dynamic compliance, *P < 0.05 between groups.

Airway pressure and lung compliance after matching are shown in Fig. 2. P_peak_ and P_plat_ showed significant intergroup differences over time (P_group*time_ = 0.027 and < 0.001, respectively). P_peak_ was consistently higher in the ETT group than in the SGA group over time and statistically significant 5 min after pneumoperitoneum (P = 0.030). P_plat_ did not show consistent trends between the two groups over time but was significantly higher in the SGA group than in the ETT group after lateral positioning (P = 0.030). C_dyn_ showed intergroup differences over time (P_group×time_ = 0.014) and was significantly higher in the SGA group than in the ETT group at all time points (all P-values < 0.05). C_stat_ did not show any significant intergroup differences (P_group×time_ = 0.171).

None of the donors showed desaturation during the surgery (SpO_2_ ≥ 97%). The EtCO_2_ was within the optimal ventilation range (≤ 45 mmHg) during surgery, except in one donor in the ETT group (EtCO_2_ 47 mmHg at 5 min after pneumoperitoneum).

The leakage fraction was investigated in all donors in the SGA group before matching (n = 205). The mean leakage fraction showed a range of 6–8% during surgery and the lateral position and pneumoperitoneum did not significantly influence the leakage fraction (after induction vs. after lateral position: P = 0.094; after induction vs. 5 min after pneumoperitoneum: P = 0.094; after lateral position vs. 5 min after pneumoperitoneum: P > 0.999). The duration of PACU stay was comparable between ETT (59 ± 11 min) and SGA (58 ± 12 min, P = 0.839) groups; hospital stays were also comparable between two groups after matching (9.0 ± 3.1 days vs. 9.0 ± 1.7 days, P = 0.868, respectively). None of the donors showed clinically significant postoperative pulmonary complications.

## Discussion

This study showed that the use of SGA during LDN was favorable in terms of respiratory mechanics based on the reduction in P_peak_ and increase in C_dyn_ compared to those achieved with the use of ETT. No cases of intraoperative desaturation (SpO_2_ < 97%) or hypercapnia (EtCO_2_ > 45 mmHg) were observed in the SGA group, suggesting adequate oxygenation and ventilation. Lateral position and pneumoperitoneum did not significantly influence the leakage fraction in the SGA group. There were no cases of postoperative pulmonary complications, particularly aspiration pneumonitis.

LDN requires simultaneous lateral positioning and pneumoperitoneum; hence, the procedure is often considered suboptimal for SGA use. Although several studies have reported the safe use of SGA in the lateral position^14^ and pneumoperitoneum^6,7^, there are only few studies on the combined effect of the lateral position and pneumoperitoneum. Rustagi et al. investigated the feasibility of LMA Proseal™, another second-generation SGA, in laparoscopic urological procedures in 25 patients^15^. They found that the mean oropharyngeal sealing pressure was always above the mean P_peak_ even in the lateral position with pneumoperitoneum. Lan et al. compared oropharyngeal sealing pressure in two different second-generation SGA (LMA Proseal™ and LMA Supreme™) in the lateral position with pneumoperitoneum and concluded that ventilation was adequate in both SGA types^16^. Our study adds to the investigation of feasibility of second-generation SGA use in the lateral position with pneumoperitoneum through analyses of respiratory mechanics and leakage fraction in a large pool of donors.

The current study suggests that mechanical ventilation using the LMA Protector™ offers more favorable respiratory mechanics than that using ETT in LDN. P_peak_ was lower during pneumoperitoneum and C_dyn_ was higher throughout surgery in the SGA group than in the ETT group. Because C_dyn_ involves combination of lung compliance and airway resistance, the results may be explained by the fact that the airway resistance under SGA was significantly lower owing to its wider inner diameter and shorter shaft compared to those of ETT^17,18^. Dynamic parameters, including P_peak_ and C_dyn_, are components of the power delivered to the respiratory system during mechanical ventilation^19^. Mechanical power is a comprehensive concept that comprises all risk factors for ventilator-induced lung injury^20,21^. Therefore, ventilation with SGA may be one of the ways to reduce ventilator-induced lung injury.

This study has several limitations. First, because of its retrospective nature, we could not determine important factors related to the safety of SGA use and airway-related complications such as oropharyngeal sealing pressure, glottis view using fiber bronchoscopy, sore throat, dysphagia, and hoarseness. Second, PaO_2_ and PaCO_2_ were replaced by measures of SpO_2_ and EtCO_2_ because intra-arterial catheters are not routinely placed in kidney donors at our institute due to their invasiveness. Third, because we used only the LMA Protector™ in the SGA group, the results may not be reproducible with other SGA devices.

In conclusion, second-generation SGA can be safe alternative to ETT for LDN. There was no change in the leakage fraction during mechanical ventilation with SGA in the lateral position or pneumoperitoneum. SGA use resulted in reduced airway resistance and increased compliance, which suggests that its use can be beneficial in the airway management of kidney donors. Our study suggests the need for a large sample size randomized study to demonstrate the safety of SGA in lateral position under laparoscopy.

## Data Availability

The datasets generated during and/or analyzed during the current study are available from the corresponding author on reasonable request.
